# Magnetic resonance imaging for deep infiltrating endometriosis: current concepts, imaging technique and key findings

**DOI:** 10.1186/s13244-021-01054-x

**Published:** 2021-07-22

**Authors:** Filomenamila Lorusso, Marco Scioscia, Dino Rubini, Amato Antonio Stabile Ianora, Doriana Scardigno, Carla Leuci, Michele De Ceglie, Angela Sardaro, Nicola Lucarelli, Arnaldo Scardapane

**Affiliations:** 1 University of Bari Medical School - Interdisciplinary Department of Medicine, Section of Diagnostic Imaging, Piazza Giulio Cesare, 11, 70124 Bari, Italy; 2 Unit of Gynecology, Mater Dei Hospital, Bari, Italy; 3 Unit of Gynecology and Obstetrics, Di Venere Hospital, Bari, Italy; 4 University of Bari Medical School - Interdisciplinary Department of Medicine, Section of Radiation Therapy, Bari, Italy

**Keywords:** Endometriosis, Deep infiltrating endometriosis, Magnetic resonance imaging, Imaging protocol

## Abstract

Endometriosis is an estrogen-dependent chronic disease affecting about 10% of reproductive-age women with symptoms like pelvic pain and infertility. Pathologically, it is defined by the presence of endometrial tissue outside the uterine cavity responsible for a chronic inflammatory process. For decades the diagnosis of endometriosis was based on surgical exploration and biopsy of pelvic lesions. However, laparoscopy is not a risk-free procedure with possible false negative diagnosis due to an underestimate of retroperitoneal structures such as ureters and nerves. For these reasons nowadays, the diagnosis of endometriosis is based on a noninvasive approach where clinical history, response to therapy and imaging play a fundamental role. Trans-vaginal ultrasound and magnetic resonance imaging are suitable for recognizing most of endometriotic lesions; nevertheless, their accuracy is strictly determined by operators’ experience and imaging technique. This review paper aims to make radiologists aware of the diagnostic possibilities of pelvic MRI and familial with the MR acquisition protocols and image interpretation for women with endometriosis.

## Key points

- Diagnostic laparoscopy is considered the gold standard for endometriosis, but it is invasive with possible false negative results.
- Nowadays there is a paradigm shift from surgical to non-invasive diagnosis based on symptoms, response to therapy and imaging.
- MRI is highly accurate for the diagnosis of Deep Infiltrating Endometriosis.
- The diagnostic results of MRI depend on an accurate imaging technique and on the comprehension of specific MR-findings.

## Background

Endometriosis, particularly deep pelvic infiltrating endometriosis (DIE), is a clinical issue affecting premenopausal women who may experience severe pelvic pain and infertility [1]. These symptoms are mainly associated with the growth of endometrial tissue outside the uterine cavity, with consequent chronic inflammatory reactions and fibromuscular hyperplasia affecting the pelvic peritoneum and the pelvic wall and organs [2]. The disease affects approximately 10% of women of reproductive age and is diagnosed in approximately 20%–50% of infertile women and nearly 90% of infertile women with chronic pelvic pain [3]. Accurate early diagnosis of DIE is crucial to provide women with early tailored treatments and avoid inappropriate surgery [4]. Nevertheless, although many diagnostic techniques have been used, early diagnosis of DIE remains a major challenge [5].

Laparoscopic exploration is considered the diagnostic golden standard, because it allows for direct visualization of lesions; however, it is not a risk-free surgery and may underestimate retroperitoneal structures, such as nerves and ureters, with possible false negative procedures [6, 7]. Because a poor correlation has been demonstrated between the symptoms and severity of lesions, some authors suggest a paradigmatic shift to a more clinical diagnostic approach based on the combination of symptoms, imaging findings and response to empiric treatment, even before any surgical confirmation [8]. In this scenario, transvaginal ultrasound, magnetic resonance imaging (MRI) and in some cases computed tomography (CT) play a fundamental role [9, 10]. MRI, the imaging technique with the highest overall accuracy for assessing the extent of DIE, has high specificity for endometriotic foci, owing to its inherent soft-tissue resolution [11, 12]. Nevertheless, to achieve the expected accuracy, the examination itself and the image interpretation should be tailored to each woman’s specific issues. This review provides radiologists with information on how to obtain good quality MRI images, interpret and report them correctly.

### Management of deep endometriosis: current concepts

Endometriosis is a complex and heterogeneous disease that may manifest with three clinical patterns with increasing severity. Superficial peritoneal lesions are characterized by superficial implants of the pelvic peritoneum; ovarian endometriomas (OMA) are hemorrhagic cysts arising from ectopic endometrial tissue growing within the ovaries and less frequently outside the ovaries [13, 14]; and DIE leads to the most severe clinical pattern and is characterized by ectopic endometrial tissue penetrating deeper than 5 mm under the peritoneal surface, thus leading to local inflammation and consequently to fibrosis and muscular hyperplasia [7, 15, 16]. DIE is usually found as a multifocal disease simultaneously involving multiple pelvic sites such as the Douglas pouch, the utero-sacral ligaments (USL), pelvic nerves, the rectum, the bladder, and the ureters [17, 18] (Table 1). The pathophysiology of such lesions is widely unknown; the type of lesion may vary over the life course, with no evidence supporting an ordered progression of endometriotic lesions [19]. Similarly, the heterogeneity of symptoms is high. Women with endometriosis may experience dysmenorrhea, dyspareunia, dysuria, constipation, chronic pelvic pain and infertility; however, a clear characterization of the pain types and topologies of implants is lacking [20]. Consequently, some women with minimal disease may report severe pelvic pain and infertility, whereas others with diffuse pelvic lesions can be almost asymptomatic [6]. The heterogeneity of the disease and the uncertainties about its pathogenesis make its diagnosis challenging. For decades, laparoscopic visualization with histologic verification of lesions was considered the golden standard for diagnosis [21]. However, laparoscopy, even if diagnostic, is not a risk-free surgery. Furthermore, deep infiltrating endometriosis may not be clearly seen in some cases during diagnosis laparoscopy [22]. In fact, the diagnosis of adenomyosis and deep endometriosis involving retroperitoneal structures, particularly ureters and nerve roots, is extremely challenging, particularly when performed by non-experienced gynecologists. False negative procedures may result, thus significantly delaying the start of appropriate management and potentially leading to major complications [7, 8]. Symptoms and clinical findings of endometriosis can result in clinical diagnoses that may be strongly supported by imaging techniques even without histological confirmation. Transvaginal ultrasound, because of its non-invasiveness, dynamicity, ease of use, availability, cost-effectiveness and reproducibility, is currently considered by many endometriosis experts as the best first-line method for assessment of DIE [4, 5, 23]. A systematic sonographic approach as defined by International Deep Endometriosis Analysis (IDEA) consensus was shown to improve detection rates of pelvic lesions [24]. MRI is also considered a highly accurate imaging modality in the evaluation of DIE, particularly when involvement of the rectum, ureters and nerve roots is suspected; these areas are highly important for both the patient and the surgeon and may be poorly visualized even with laparoscopy [25, 26]. MRI is also increasingly used to assess the anatomic response to medical or surgical treatment and to differentiate endometriosis from adenomyosis; the latter is a specific and heterogeneous disease contributing, independently of endometriosis to symptoms, defined as the invasion of endometrial tissue into the myometrium occurring in different forms (diffuse, focal, cystic or superficial). Adenomyosis may exist on its own but in about 30% of cases it is associated with DIE [7, 27]. The shift toward clinical and imaging-based diagnosis shortens the time between the first consultation and the final diagnosis [28], but the use of non-invasive methods requires a rigorous approach to ensure meaningful and consistent results [8, 29].

**Table 1:** Most frequent pelvic localization of DIE

Site	Frequency (%)
Pouch of Douglas/retrocervical	55–60
Uterosacral ligaments	32–57
Recto-vaginal septum	20–48
Bowel (overall) Rectum Recto-sigmoid junction Sigmoid Cecum/appendix Small bowel	16–35 30–40 25–30 15–20 5 5
Bladder	5–8
Sacral nerves	3–5

References no. [12, 18, 37, 46, 53]

### MRI acquisition protocol: dos, don’ts and maybes

MRI is a widely used technique for the diagnosis of DIE; however, an international consensus on the best imaging protocol is lacking. Recent guidelines published by the European Society of Urogenital Radiology describe consensus suggestions from a conference among nine imaging centers in Europe and one in Japan [29]; however, the indications and imaging protocols may vary among institutions according to local expertise. In general, when suspicion of endometriosis exists, MRI should be used first to provide an adequate anatomic representation of the entire pelvis and its organs and second to ensure the recognition of DIE, according to the contrast between normal pelvic fatty tissue and endometriotic lesions or on the detection of hemorrhagic cysts and foci (Table 2).

**Table 2:** Standard MRI protocol for endometriosis in our center

Sequence	Plane	Voxel mm (AP-RL-thickness)	FOV (mm)	NEX	TE
TSE T2	Axial/Obl axial	0.9–0.9–3	280–350	2	100
TSE T2	Sagittal	0.9–0.9–3	180–250	2	100
TSE T2	Coronal	0.8–0.8–3	280–300	2	100
TSE T1	Axial	0.9–0.9–3	280–350	1	Shortest
THRIVE	Axial	0.75–0.75–3	280–350	3	Shortest
THRIVE	Sagittal	0.75–0.75–3	280–350	3	Shortest
*Optional sequences*					
CE-THRIVE	Axial/Sagittal	0.75–0.75–3	280–350	3	Shortest
BTFE*	Axial/sagittal	1.5–1.5–4	280–350	1	Shortest
SSFSE T2*	Axial/sagittal	1–1–4	280–350	1	100

^*^Performed with rectal distension

### DOS

**Fig. 1:**
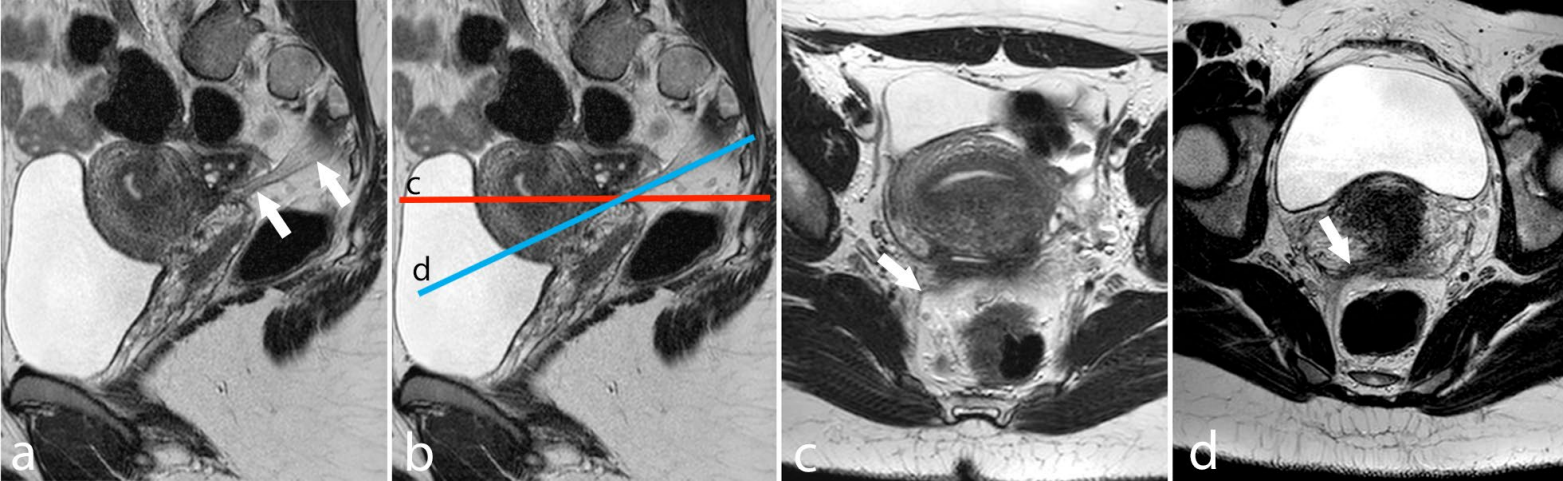
Mild thickening of the right USL in a woman with DIE. **a**–**b** T2w sagittal images. **c** T2w axial image obtained along the red plane shown in **b**. **d** T2w oblique axial image obtained along the blue plane shown in **b**. The USL produces a better depiction of the sagittal and oblique axial plane (arrows)

MRI for endometriosis should be performed with a 1.5 T or 3 T scanner and high-resolution phased array coils (with 8–16 channels), whereas low-magnetic field or open-MRI lacks sufficient image quality to image DIE. High-resolution, thin section (3 mm) TSE-T2w sequences in the sagittal, axial and coronal planes are crucial to evaluate DIE, whereas TSE T1w (with and without fat saturation) should always be obtained to depict adnexal hemorrhagic lesions such as OMA. Oblique planes may be highly useful to visualize specific anatomical structures such as utero-sacral ligaments (Fig. 1) [30]. A substantial improvement in image quality may be obtained by using rectal cleansing and anti-peristaltic agents such as butyl-scopolamine or glucagon which can also be helpful in the evaluation of adenomyosis. Some authors suggest a more reliable effect of such agents when intravenous rather than intramuscular injection is used; however, intramuscular administration ensures longer anti-peristaltic results, in line with an average imaging duration of 20–25 min [31]. The pelvis should be imaged regardless of the phase of the menstrual cycle, in patients with a moderately full bladder [29].

#### DON’Ts

**Fig. 2:**
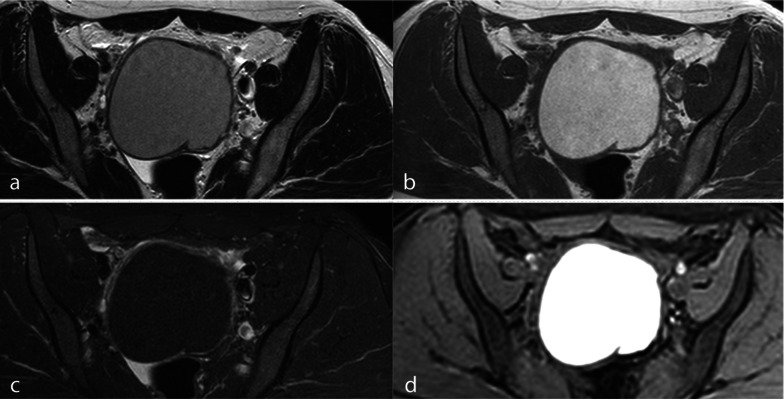
OMA mimicking a mature cystic teratoma in a STIR sequence. **a** TSE T2w axial image, (**b**) TSE T1w axial image, (**c**) STIR axial image and (**d**) THRIVE axial image. The loss of T1w high signal intensity in the STIR image is not specific to fat (**c**), because endometriomas and fatty tissue may have similar T1 relaxation times. In the THRIVE sequence, on the basis of a spectral saturation of fat, the endometrioma remains hyperintense (**d**)

Because the recognition of DIE is based on the contrast between the high signal intensity of fatty tissue and low signal intensity of endometriotic nodules, fat-saturated T2w images should not be used [32, 33]. Among T1w fat-saturated techniques, STIR sequences should be avoided. These sequences, which are based on the T1 relaxation time, yield a non-specific saturation, which may suppress nonfatty tissues with similar T1 values, such as the blood when methemoglobin is present, thus leading to a difficult differential diagnosis between mature cystic teratomas and endometriomas (Fig. 2) [34, 35]. Selective saturation of fatty tissue can be obtained with spectral saturation (SPAIR or SPIR) used in 2D SPIR T1 images or in 3D interpolated sequences, such as THRIVE or DIXON.

### MAYBES

The use of intravenous contrast media is widely debated in the literature. Deep endometriosis is recognized by a low signal intensity tissue with small hyperintense foci in T2w images, which may also show distortion of the pelvic anatomy associated with adhesions. Therefore, contrast-enhanced (CE) images appear to be useless in the diagnosis of DIE. However, for specific indications, the injection of Gd-based contrast agent may be advisable. CE-images are mandatory in cases of complex adnexal hemorrhagic cysts showing mural thickening or other potentially malignant features in T2w images. Similarly, the use of contrast agents may aid in differentiating endometriomas from luteal ovarian cysts or tubo-ovarian abscesses [36, 37]. In our center, we have found that combining MR colonography with CE THRIVE images may enable the diagnosis of colorectal involvement by less experienced radiologists thanks to an easier recognition of thickened wall and for the possibility to distinguish enhancing nodules from endoluminal fecal material or air [38]. Post-contrast MR urography should be used when the ureteral involvement is suspected to define the degree of urinary tract dilation and the precise site of infiltration [39].

No consensus exists in the literature regarding the usefulness of vaginal and rectal opacification for the diagnosis of DIE; some authors find them extremely useful and have proposed the use double contrast barium enema or cross-sectional colonography with either CT or MRI [40–42], whereas others have reported no diagnostic improvement from these procedures [43]. In our center, we use rectal distension in patients showing an endometriotic lesion infiltrating the rectum in standard TSE T2w images to quantify the stenosis, which according to our experience is predictive of the need for bowel resection [25]. However, several alternative methods based on T2w images without rectal distension have been described to predict the need for bowel resection [44, 45].

### MR anatomic landmarks

**Fig. 3:**
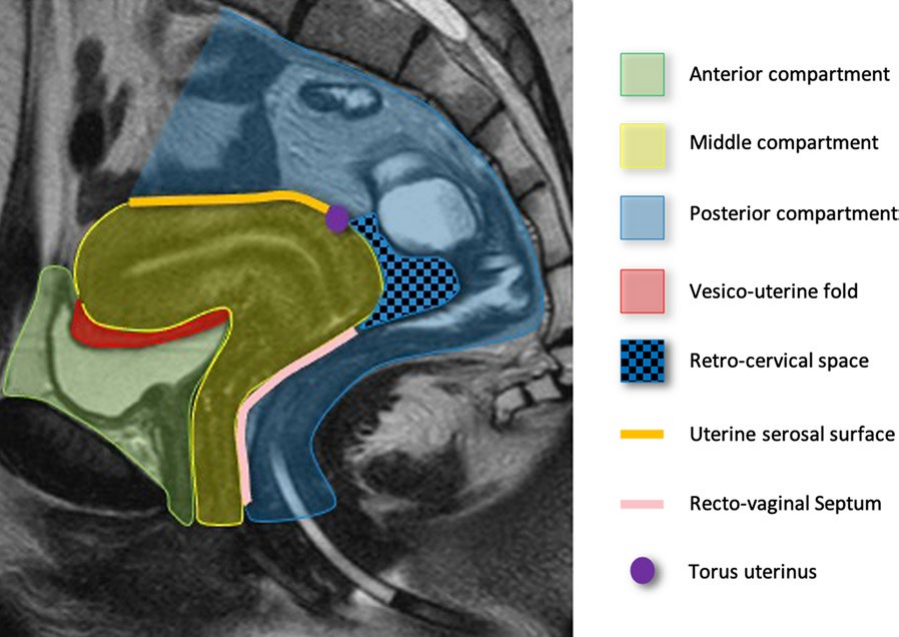
Midsagittal T2w image of female pelvis with main anatomic landmarks to be considered in the evaluation of DIE

The imaging evaluation of endometriosis should be guided by the statistical frequency of involvement of the pelvic anatomy [11] and be consistently accurate. To achieve a uniform evaluation of women with suspected endometriosis, the IDEA group in 2016 and the society of Abdominal Radiology have proposed a consensus lexicon for reporting US and MRI, respectively [24, 46]. In both experiences, it is suggested to report findings by pelvic compartments (anterior, middle and posterior) and using consistent anatomic landmarks (Fig. 1, 3).

Anterior compartment is the space limited anteriorly by the pubic symphysis and posteriorly by the uterus and contains the urinary bladder, the vesico-uterine fold and the round ligaments. The middle compartment contains the uterus and the ovaries, while the posterior compartment can be divided into the recto-uterine, recto-cervical spaces and the recto-vaginal septum and contains the serosal surface of the uterus, the pouch of Douglas, the torus uterinus, the USL well as the rectum and the sigmoid colon.

### MRI findings

Endometriosis is a multifocal disease that may involve multiple pelvic structures with possible extra-pelvic extension. OMA, superficial peritoneal lesions, and DIE have been reported in surgical series studies to affect the ovaries in 65–80%, 45–50%, and 63–70% of women, respectively [12, 47, 48]. DIE is usually more frequent in the posterior pelvic compartment (95% of cases) including the torus uterinus, the recto-vaginal septum, USL, pouch of Douglas and anterior wall of the rectum than the anterior pelvic compartment (including the vesico-uterine pouch and bladder; 16% of cases). Both compartments may be involved in approximately 10% of cases, whereas ureter and nerve lesions are seen in 5% of patients [47, 49].

**Fig. 4:**
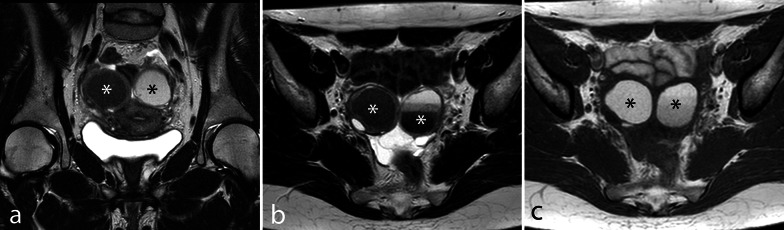
Bilateral OMA. TSE T2 coronal (**a**) and axial (**b**) images and TSE T1w axial image (**c**). Bilateral endometriomas; the left-sided endometrioma shows a stratified aspect in b (shading sign). Of note, the ovaries are prolapsed in the pouch of Douglas, touching each other at the midline (kissing ovary sign)

**Fig. 5:**
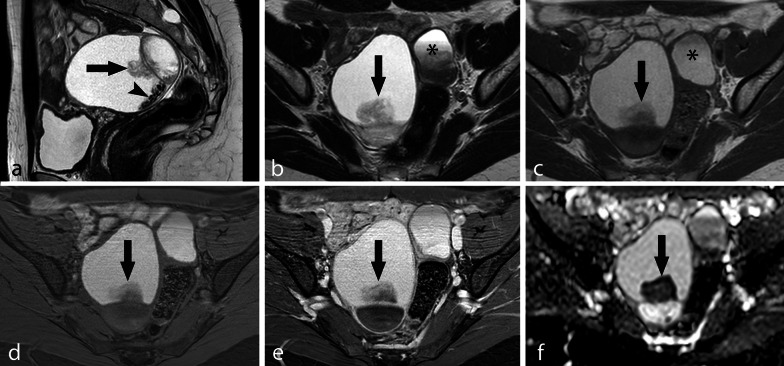
Large OMA with irregular mural vegetation and dark spots. TSE T2w sagittal image (**a**), TSE T2w axial image (**b**), TSE T1w axial image (**c**), axial THRIVE image (**d**), contrast-enhanced axial THRIVE image (**e**) and ADC map (**f**). A large right multiloculated OMA is shown with an irregular mural vegetation (arrow) and small dark spots (arrowhead). The vegetation shows no significant contrast enhancement (**e**) but restricted diffusion (**f**) and should be considered suspected for malignancy. A smaller left-sided OMA with shading sign is also visible (*)

OMA may manifest as solitary or multiple thick-walled cysts showing homogeneous high signal intensity in T1w and fat-saturated T1w images regardless of the intensity in T2w images. According to the age at bleeding onset, OMA may be either hyperintense or hypointense in T2w images or may show a typical stratified appearance (shading sign), as a result of cyclic bleeding with blood products accumulating over the course of months (Fig. 4) [37]. In some cases, dark spots (low-intensity, well-defined images in T2w sequences) may be visible within cysts (Fig. 5) [50]. Irregular mural thickenings or mural vegetations should be studied after the intravenous injection of contrast agents and DWI sequences to exclude malignant transformation (Fig. 5).

**Fig. 6:**
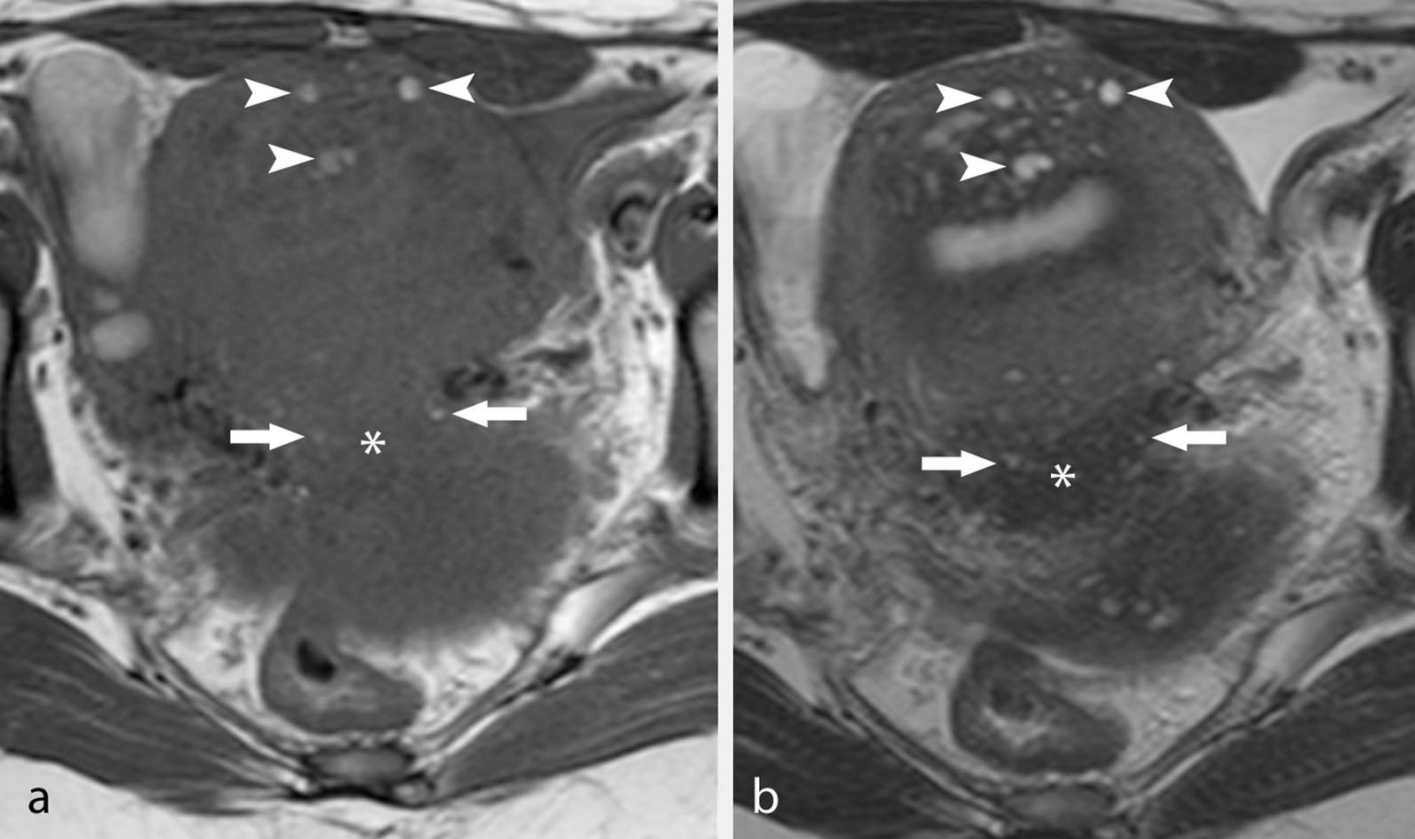
Retro-uterine DIE nodule. TSE T1w image and TSE T2w image. DIE nodules are characterized by intermediate signal intensity in T1w images (* in **a**) and low signal intensity in T2w images (* in **b**), with high intensity foci in both sequences (arrows). Adenomyosis is also shown with a similar MR aspect within the anterior wall of the uterus (arrowheads)

**Fig. 7:**
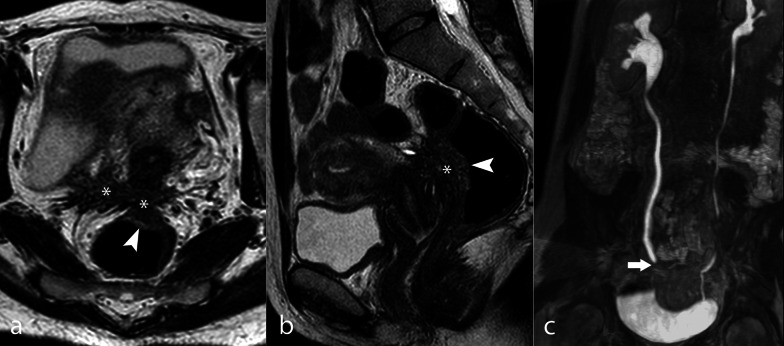
Severe DIE with ureteral involvement. TSE T2w axial image (**a**), TSE T2w sagittal image (**b**) and CE MR urography (**c**). Large DIE nodule of the pouch of Douglas extending to the right USL (*) and the anterior rectal wall (arrowhead). MR urography shows the involvement of the right pelvic ureter (arrow), with consequent moderate hydronephrosis

DIE can manifest as pelvic nodules or plaque-like lesions and adhesions [51, 52]. Nodules and plaque-like lesions are composed of endometrial glands and stroma surrounded by a thick fibro-muscular and inflammatory reaction, and usually have an irregular, spiculated shape and a signal intensity similar to that of pelvic muscles, with intermediate signal intensity in T1w sequences and low signal intensity in T2w images. Small hyperintense foci corresponding to endometrial glands are almost always recognized within the endometriotic nodules in both T1w and T2w images (Fig. 6). The most common site of DIE nodules is the posterior pelvic compartment, where all anatomic structures bordering the pouch of Douglas can be involved (the posterior border of the cervix, the torus uterinus, the uterosacral ligaments, the vaginal wall, the anterior wall of the rectum and the recto-sigmoid junction; Fig. 7a, b) [53].

**Fig. 8:**
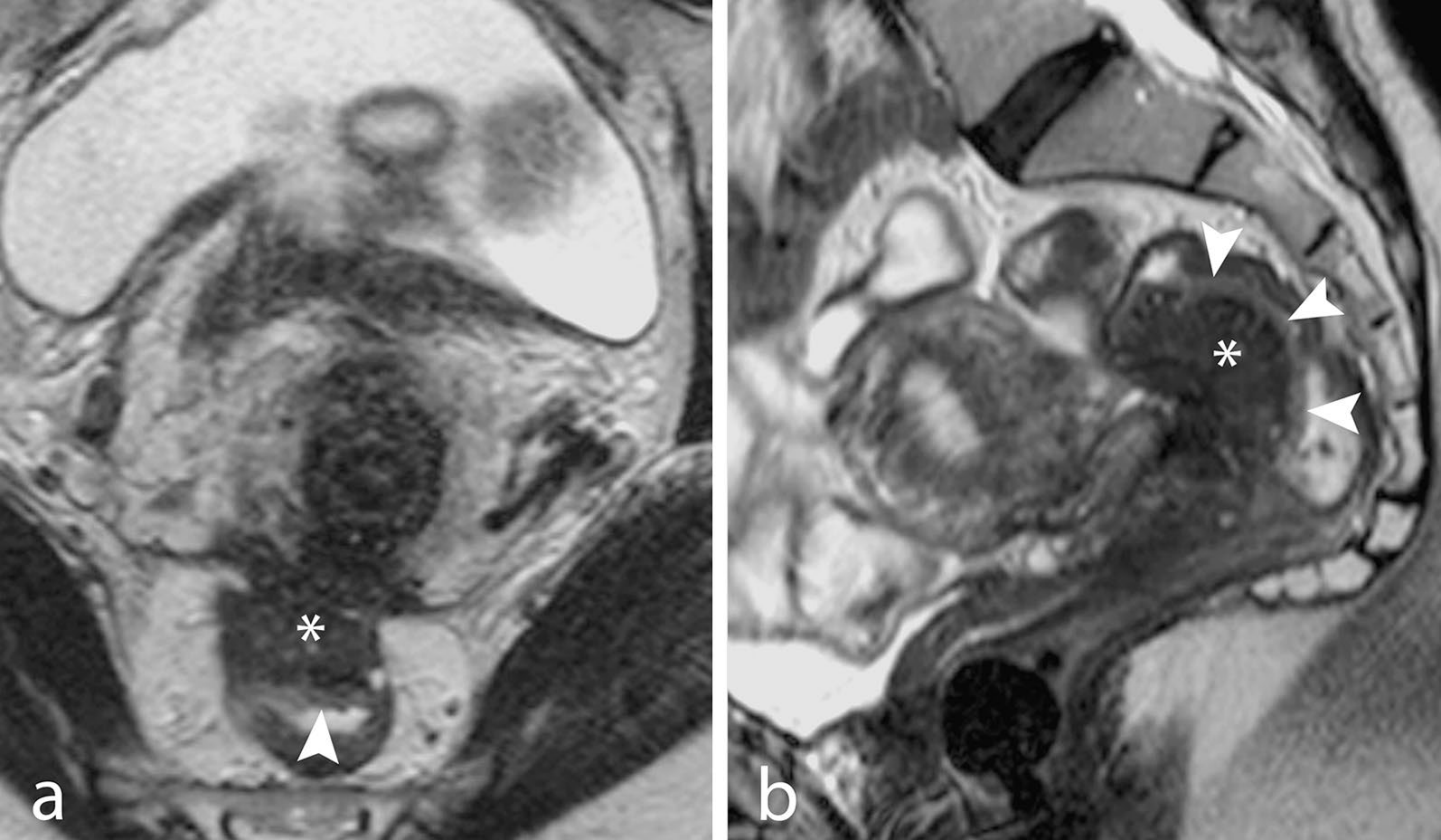
DIE with rectal infiltration. TSE T2w axial (**a**) and sagittal (**b**) images. A large DIE nodule (*) infiltrates the anterior wall of the rectum. The nodule has a mushroom-cap shape with a bright peripheral rim (arrowhead) corresponding to a normal mucosa layer

**Fig. 9:**
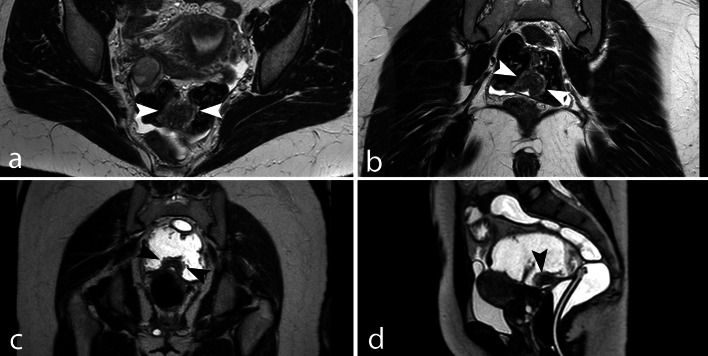
DIE with cecal infiltration. TSE T2w axial (**a**) and coronal (**b**) images, SSFSE T2 MR-colonography coronal (**c**) and sagittal (**d**) images. A large DIE nodule with a mushroom shape (arrowheads) infiltrates the cecum which has a pelvic position in the pouch of Douglas

Diagnosis of bowel involvement is based on the presence of a nodular or plaque-like endometriotic bowel wall thickening and loss of the fat tissue plane between the intestinal loop and the uterus or other adjacent organs. The most frequent sites of bowel endometriosis are the rectum and the sigmoid colon, while the involvement of the cecum or the ileum can be found in about 5% cases (Table 1). The diagnosis may be facilitated by the presence of ancillary findings such as a “mushroom cap” sign (Figs. 8, 9) [54]. This sign can be visible in any of the plane of the space and represents the endometriotic nodule growing into a mushroom-like shape in the bowel wall, covered by a high intensity signal rim representing the normal mucosa and submucosal layer (Figs. 7, 8, 9).

**Fig. 10:**
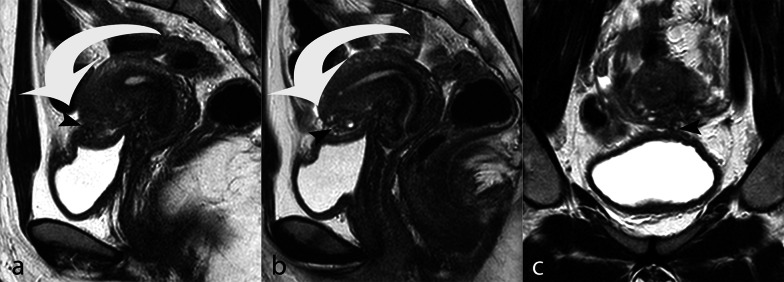
DIE with vesical infiltration. TSE T2w sagittal (**a**, **b**) and coronal (**c**) images. The vesico-uterine fold is occupied by a DIE nodule (arrowhead) anteriorly tethering the uterine body (curved arrow)

**Fig. 11:**
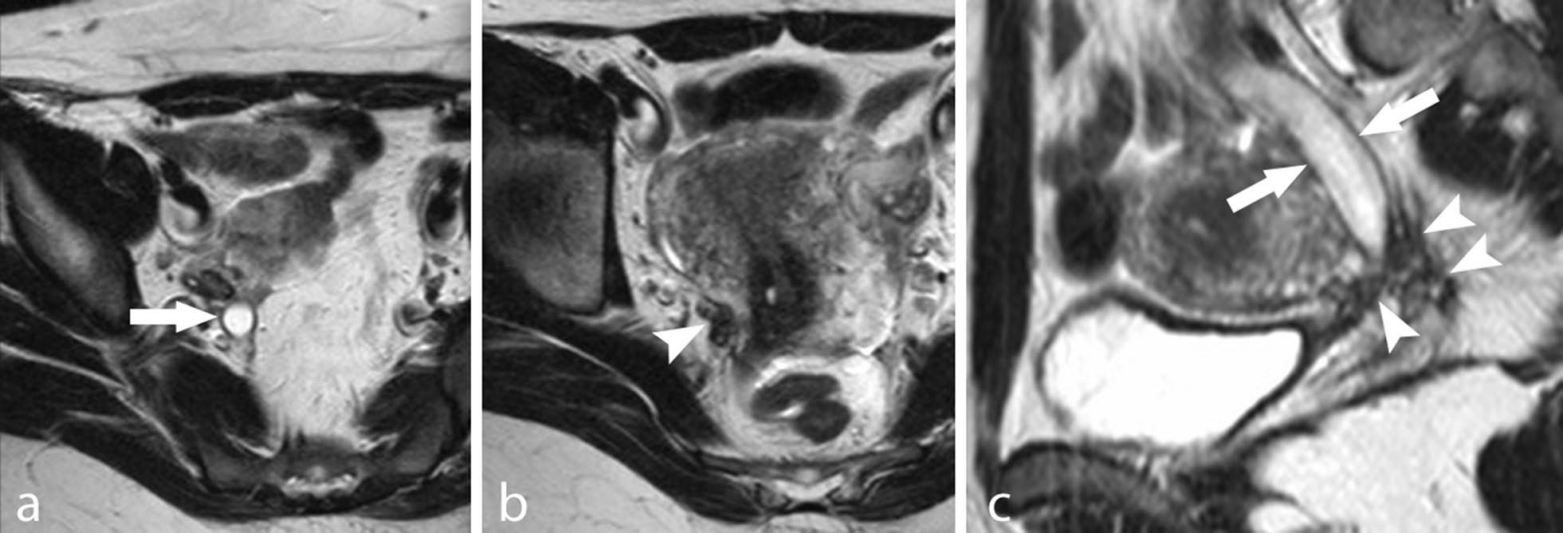
DIE with right ureter infiltration. TSE T2w axial (**a**, **b**) and sagittal (**c**) images. A right para-uterine DIE nodule (arrowheads) infiltrates the distal tract of the right ureter, which is dilated (arrows)

Endometriotic nodules of the anterior or lateral pelvic compartment are less frequently observed and usually involve the urinary system, particularly the vesical dome for nodules of the vesico-uterine fold (Fig. 10) and the ureters for lesions extending in the para-vesical space (Fig. 11). Axial and sagittal TSE T2w images are the most sensitive in identifying ureteral nodules; however, in these cases, the examination should be completed with post-contrast MR urography to demonstrate even mild urinary dilatation and the exact position of ureteral involvement (Fig. 7c).

**Fig. 12:**
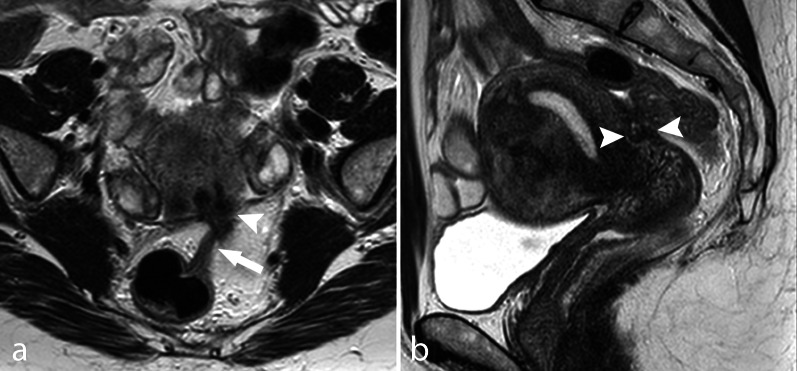
Adhesive obliteration of the pouch of Douglas. TSE T2w axial (**a**) and sagittal (**b**) images. The anterior rectal wall is tethered (arrow) to the posterior surface of the uterus, where a DIE nodule is seen (arrowhead)

**Fig. 13:**
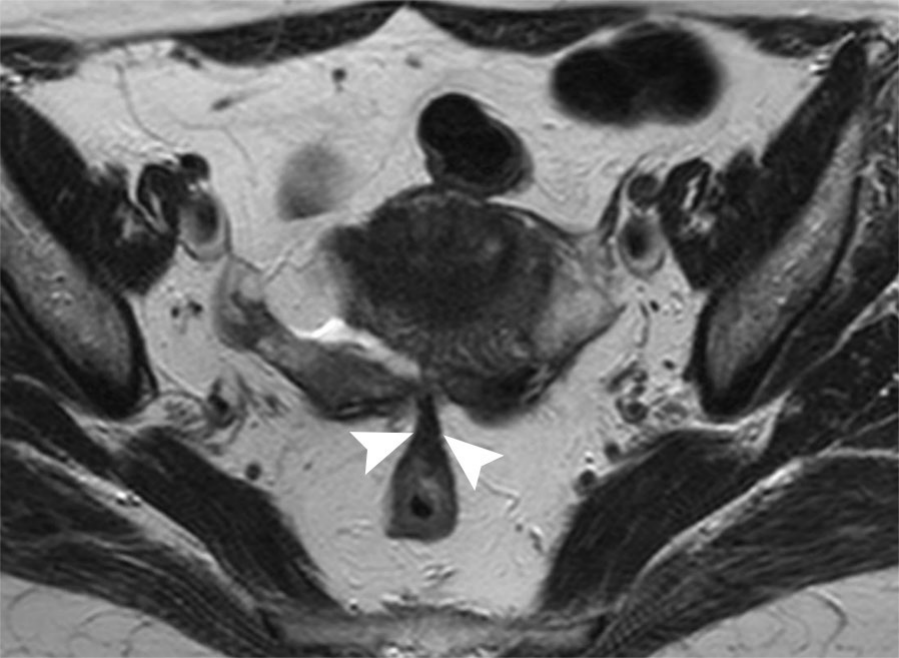
DIE of the pouch of Douglas. TSE T2w axial image. Teardrop deformation of the rectum for DIE adhesions (arrowheads) is seen

In many cases, MRI may depict pelvic changes consistent with the presence of adhesions, which indirectly suggest DIE. In general, adhesions are suspected when fatty interfaces between adjacent structures are not clearly visible in any orthogonal planes. The most reliable finding to diagnose endometriotic adhesions is tethering and angulation of normal pelvic structures and bowel loops (Fig. 12). Adhesion between the anterior wall of the rectum and the posterior surface of the uterus, with a consequent “teardrop” deformation of the rectum and retroversion of the uterine body, is frequently seen in pelvic MRI and is specific for DIE (Fig. 13). Similarly, ovaries may prolapse in the Douglas pouch and create adhesions between each other and the uterine wall on the midline, thus producing a so-called kissing ovary sign, which is a common finding in DIE of the posterior pelvis (Fig. 4). Douglas obliteration should be suspected when nodules extend from the retro-cervical space to the anterior wall of the rectum or when adhesions are seen at this level (Figs. 7, 8, 12). In contrast, if small bowel loops are seen between the uterus and the rectum, the obliteration of the pouch of Douglas can be ruled out [53].

**Fig. 14:**
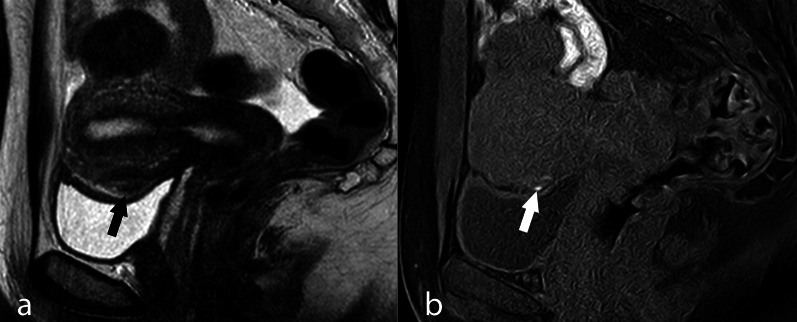
Adhesive endometriosis of the vesico-uterine pouch. TSE T2w (**a**) and THRIVE (**b**) sagittal image. Small hyperintense spot-like images of the vesico-uterine pouch are seen. An endometriotic plaque was found through laparoscopy

Anterior pelvic adhesions usually occur between the uterus and the bladder for plaque-like or linear implants in the vesico-uterine pouch, which may be visible as small spots with high signal intensity in the sagittal T1w fat-saturated images (Fig. 14) but can nonetheless be easily missed by pelvic MRI, whereas endo-vaginal US, owing to its ability to show an absence of sliding of the uterus along the bladder surface, is by far more sensitive.

**Fig. 15:**
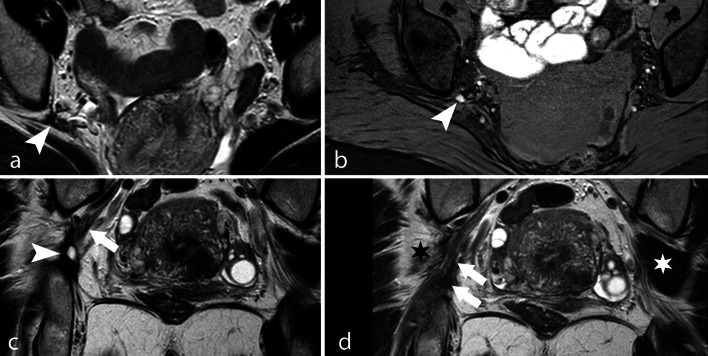
Neural endometriosis. TSE T2w axial image (**a**), THRIVE axial image (**b**) and TSE T2w coronal images (**c**, **d**). A DIE nodule of the ischiatic foramen (arrowhead) surrounds the ischiatic nerve (arrows). Note the atrophy of the right piriformis muscle (black star) and normal left piriformis muscle (white star)

Neural endometriosis is a rare condition characterized by perimenstrual radicular pain with no evidence of any alteration of the lumbar spine. The most affected nerves are the sacral plexus (57% of cases) and the sciatic nerve (39% of cases) [49, 55]. MRI is the method of choice for the diagnosis of neural endometriosis, because transvaginal ultrasound cannot depict this anatomic area. The diagnosis relies on the recognition of endometriotic nodules along pelvic nerves and on indirect signs such as denervation muscular atrophy of the affected site (Fig. 15).

Adenomyosis in 30% of cases is associated with DIE; the presence of ill-defined nodules with hyperintense foci within uterine wall in T1w and T2w images or a thickening of the junctional zone > 12 mm, is the most common findings of this specific condition (Fig. 6); however, the description of adenomyosis is beyond the purpose of this paper and is detailed elsewhere [27, 56].

## Conclusion

Because of its extreme clinical heterogeneity, pelvic endometriosis remains challenging to diagnose. Current evidence demonstrates that the disease should be diagnosed non-invasively by combining the information from patient history, clinical examination, imaging and response to medical treatment [7]. Because the diagnostic accuracy of MRI may differ depending on radiologist experience, this review article aims to help radiologists obtain meaningful images with a tailored MR-acquisition protocol and recognize a wide range of pelvic changes that may result from endometriosis.

## Data Availability

The datasets used and/or analyses during the current study are available from the corresponding author on reasonable request.

## Abbreviations

BTFE: Balanced turbo field echo
CE: Contrast enhanced
CT: Computed tomography
DIE: Deep pelvic infiltrating endometriosis
ESUR: European society of urogenital radiology
MRI: Magnetic resonance imaging
NE: Neural endometriosis
OMA: Ovarian endometrioma
RVS: Recto-vaginal septum
SSFSE: Single shot fast spin echo
SUP: Superficial peritoneal lesions
THRIVE: T1 high-resolution volume
TSE: Turbo spin echo
TVUS: Transvaginal ultrasound
USL: Utero-sacral ligaments
